# Strategies for integrating ChatGPT and generative AI into clinical studies

**DOI:** 10.1007/s44313-024-00045-3

**Published:** 2024-12-24

**Authors:** Jeong-Moo Lee

**Affiliations:** Department of Surgery, Division of HBP Surgery, Seoul National University Hospital, Seoul National University College of Medicine, 101 Daehak-ro, Jongno-Gu, Seoul, 03080 Republic of Korea

**Keywords:** Generative AI, Clinical Research, ChatGPT, Research Methodology, Academic Writing

## Abstract

Large language models, specifically ChatGPT, are revolutionizing clinical research by improving content creation and providing specific useful features. These technologies can transform clinical research, including data collection, analysis, interpretation, and results sharing. However, integrating these technologies into the academic writing workflow poses significant challenges. In this review, I investigated the integration of large-language model-based AI tools into clinical research, focusing on practical implementation strategies and addressing the ethical considerations associated with their use. Additionally, I provide examples of the safe and sound use of generative AI in clinical research and emphasize the need to ensure that AI-generated outputs are reliable and valid in scholarly writing settings. In conclusion, large language models are a powerful tool for organizing and expressing ideas efficiently; however, they have limitations. Writing an academic paper requires critical analysis and intellectual input from the authors. Moreover, AI-generated text must be carefully reviewed to reflect the authors’ insights. These AI tools significantly enhance the efficiency of repetitive research tasks, although challenges related to plagiarism detection and ethical use persist.

## Introduction

The introduction of ChatGPT has resulted in significant changes in various sectors of society [1, 2]. A generative pre-trained transformer (GPT) is an artificial intelligence model designed to emulate human language by learning from large amounts of textual data [3]. The launch of ChatGPT as a chatbot in late 2019 generated considerable interest and impact [4]. The general population has begun to use this technology across diverse fields, with applications spanning virtually every aspect of society. Owing to its remarkable efficiency in enhancing productivity, ChatGPT rapidly collected a large user base. Concurrently, owing to substantial investments from Microsoft, OpenAI, the creator of ChatGPT, continues to lead the field as a pioneer in this domain [5].

Recently, Claude, which was developed by Anthropic, demonstrated impressive capabilities [6]. Additionally, various models, such as Perplexity, which address several limitations of ChatGPT, have entered the market [7].

Despite these advances, the use of ChatGPT in the medical field remains a subject of considerable debate [8, 9]. The initial hallucinations exhibited by GPT-3 created significant obstacles for many potential users. Instances of fabricating non-existent research papers or providing responses based on outdated knowledge without accurate information retrieval were common. In certain cases, this led to the publication of papers by researchers who indiscriminately used AI tools [10, 11].

Due to such incidents, several journals denied accepting papers that utilized generative AI. In response to these recurring issues, the International Committee of Medical Journal Editors issued guidelines for using ChatGPT and other generative AI in research activities [12]. The key points of these guidelines include using AI primarily to enhance readability and disclosing the use of generative AI, and they stipulate that generative AI cannot be credited as an author [13]. Many medical journals quickly adopted these guidelines, and numerous journals now include an "AI writing" section in their author guidelines detailing the proper use of generative AI [14–17].

Large language models (LLMs) have made remarkable progress over the past two years, significantly improving their capabilities and reducing the occurrence of hallucinations. Furthermore, GPT, which began as a natural language model, has evolved into a multimodal model capable of recognizing text, sounds, images, and videos [18]. This expansion has led to increased multifunctionality, prompting a shift in focus from regulation to considering how to use these tools effectively and responsibly.

This review explores how generative AI can be applied in medical research, how it can enhance productivity, and, most importantly, how it can be used ethically and responsibly through various examples.

### Overview of ChatGPT and generative AI development

ChatGPT, launched by OpenAI, is the most widely used LLM service. By subscribing to the paid version, users can access the high-performance GPT-4o model, which exhibits excellent performance in various areas, such as data analysis and document writing. ChatGPT's Code Interpreter has become an essential tool for automating data tasks such as code execution, statistical analysis, and data visualization. This tool allows researchers to efficiently process and analyze large datasets with minimal manual coding. In July 2023, the Code Interpreter was renamed Advanced Data Analysis (ADA), reflecting its enhanced capabilities. The ADA now supports more complex data analysis tasks, including handling large datasets and performing advanced statistical modeling. Moreover, it is integrated with platforms such as Google Drive and Microsoft OneDrive for easy data access. ADA is a default feature in GPT-4o, available to all users of ChatGPT, including free subscribers. The ability of ADA to work with various file formats and generate interactive charts simplifies the research process. Researchers can quickly produce detailed reports and visualizations, improving data analysis speed and accuracy.

Claude 3.5, developed by Anthropic, performs well as a competitor. The Artifact function of Claude was introduced as part of the Claude 3.5 Sonnet model in June 2024. Initially available as an experimental feature, it became generally accessible in August 2024. Artifacts allow users to create self-contained outputs such as code snippets, text documents, websites, and visual diagrams. These outputs are designed to be reusable and can be modified and refined outside the chat interface, providing users a more collaborative and creative environment.

Furthermore, Google's Gemini Pro emphasizes integration with services such as Google Search, Google Docs, Google Slides, and CoLab. Engineers who have previously worked at OpenAI, Google, and Meta have independently developed Perplexity AI, which offers a Retrieval Augmented Generation (RAG) service to minimize the hallucinations of traditional generative AI. This allows academic writing based on references without hallucinations. In collaboration with OpenAI, Microsoft offers the 365 Copilot service that operates within Word and PowerPoint. These models can be used in research depending on their specific functions.

### Applications of ChatGPT and LLM in medical research

#### Pre-research step

1. Brainstorming and Ideation for Research

Relevant ideas can be generated by analyzing keywords or questions pertinent to a specific topic. This capability enables researchers to select topics more efficiently and formulate research plans for scholarly articles. This serves as an invaluable brainstorming tool for researchers such as PhD candidates, research fellows, and junior staff who require publication achievements.

LLMs, particularly those like ChatGPT, can significantly enhance the brainstorming and ideation phases of medical research. These are key ways these tools can be used.

#### Generating research questions

Researchers can prompt AI to suggest potential research questions based on current trends or gaps in the field. For example:

- Prompt: "What are unexplored areas in the relationship between gut microbiome and neurodegenerative diseases?"

#### Exploring interdisciplinary connections

AI can help identify potential links between different medical fields or even seemingly unrelated disciplines.

- Prompt: "How might concepts from fluid dynamics apply to understanding blood flow in small vessels?"

#### Hypothesis generation

Researchers can use AI to generate multiple hypotheses for a given research question.

- Prompt: "Generate five possible hypotheses explaining the observed increase in autoimmune diseases in urban areas."

#### Identifying research trends

AI can analyze recent publications to identify emerging trends or shifts in research focus.

- Prompt: "What are the current trending topics in oncology research over the past 2 years?"

#### Methodological brainstorming

AI can suggest various research methodologies or experimental designs:

- Prompt: "Propose three different study designs to investigate the long-term effects of intermittent fasting on cardiovascular health."

#### Ethical consideration exploration

AI can help brainstorm potential ethical issues related to the proposed research.

- Prompt: "What are potential ethical concerns in a study using AI for early Alzheimer's diagnosis?”

#### Literature gap analysis

AI can assist in identifying gaps in existing literature:

- Prompt: "What aspects of COVID-19's impact on mental health are understudied based on current literature?"

Although AI-assisted brainstorming techniques are powerful, it is crucial to remember several key points.

- AI-generated ideas should be the starting point for further exploration and critical evaluation by researchers.
- Thoroughly verifying and evaluating the thoughts or hypotheses provided by AI using reliable sources is essential.
- Researchers should be transparent regarding using AI when submitting proposals or manuscripts during the ideation process.
- AI should complement, not replace, human creativity and expertise in the research-ideation process.

Using LLMs such as ChatGPT in this manner, researchers can potentially accelerate the process of developing novel and impactful research ideas while ensuring a comprehensive exploration of the research landscape.

2. Literature review

Researching existing studies is crucial during the pre-research-planning stage. To conduct meaningful research, it is necessary to analyze existing studies, identify their limitations and shortcomings, and develop new research plans. In the past, researchers typically used books in libraries. They manually organized the main parts or copied and organized them manually. The development of web browsers and word processors has enabled anyone to access PubMed.gov and the Cochrane Library to search for relevant literature. However, the amount of information available has increased exponentially, as has the amount of work required to organize it. Researchers often expend a significant amount of energy in literature searches, leading to insufficient energy during the actual research process.

The implementation of AI has the potential to transform the process of searching, summarizing, and organizing research, making it more innovative. GPT has recently introduced a service designated as GPTs, which performs specific functions. The combination of services, such as Consensus, which specializes in searching for research articles, with GPTs can potentially result in a synergistic effect. Instead of searching Google Scholar or PubMed, researchers can use this platform to obtain the results of their search and answers to their queries with references to relevant papers.

Even in the recently updated GPTs 4 and 4o, asking relevant questions is better than before; however, there are still cases of hallucinations, which can lead to more time spent on fact-checking. Using these GPTs (Scholar GPT, Consensus, Scholar AI, and Scispace), which are specialized for research purposes, accurate and informed answers can be obtained.

Furthermore, services such as SciSpace can be used to provide lists of the articles that were searched. Rather than reading the full text or abstracts, users receive a summary of articles that have been organized by insights, methods, results, limitations, and other pertinent information. This summary is designed to facilitate a more expedient understanding of the subject matter, facilitating a more efficient organization of research than would be possible through keyword searches in PubMed and the exploitation of the full text of articles. Furthermore, the Ask Copilot feature enables users to inquire about selected articles, allowing them to request additional information about a particular pivotal article or explore the desired section.

However, these tools have limitations. They are open-source database (DB)-centered services, such as arXiv, meaning they cannot access articles in closed, paid databases. Consequently, if an article is not included in the queried database, AI-generated responses may be inaccurate or incomplete, leading to hallucinations. Hallucinations in LLMs often arise when there is insufficient data for reference, particularly in open-source databases.

To prevent this situation, it is recommended that the GPT uses the RAG method, which incorporates the most recent search content. RAG, or search-based generation, enables GPT to organize the latest DB through an initial search rather than relying solely on its existing knowledge, directing it toward an answer within that DB. Users with coding expertise can implement RAG using the API, and the Perplexity AI platform offers a solution that provides this capability.

### Research procedure step

1. Enhancing academic writing through AI-language models

The application of AI language models has the potential to enhance the quality and clarity of academic writing, particularly in the medical field. These advanced systems can analyze researchers' drafts and generate coherent sentences and paragraphs, enriching the content and improving overall readability.

Furthermore, AI language models offer customization options for aligning text with specific requirements. Users can adjust parameters such as.

- Tone: Requesting "professional" or "explanatory" styles to suit the intended audience and purpose.
- Dialect: Selecting between British or American English to ensure consistency.
- Sentence structure: Modifying sentence length and complexity through prompts such as "make longer" or "make more concise."

2) Streamlining research documentation with AI-language models

The documentation process in academic research often poses a significant challenge, potentially causing delays in the overall research progress. AI language models, particularly LLMs such as GPT, have emerged as powerful tools for addressing the often challenging and time-consuming research documentation process.

These AI systems can assist researchers in preparing essential documents throughout their research workflow, including research proposals, ethical approval applications, progress reports, grant applications, and manuscript chapters. Researchers can accelerate the documentation process by leveraging AI language models, leading to increased efficiency and improved quality.

The following is an example of creating a document based on the study title, informed consent, case report form, an Excel file for data collection, and an E-CRF page in HTML language for the study.

**Fig. 1:**
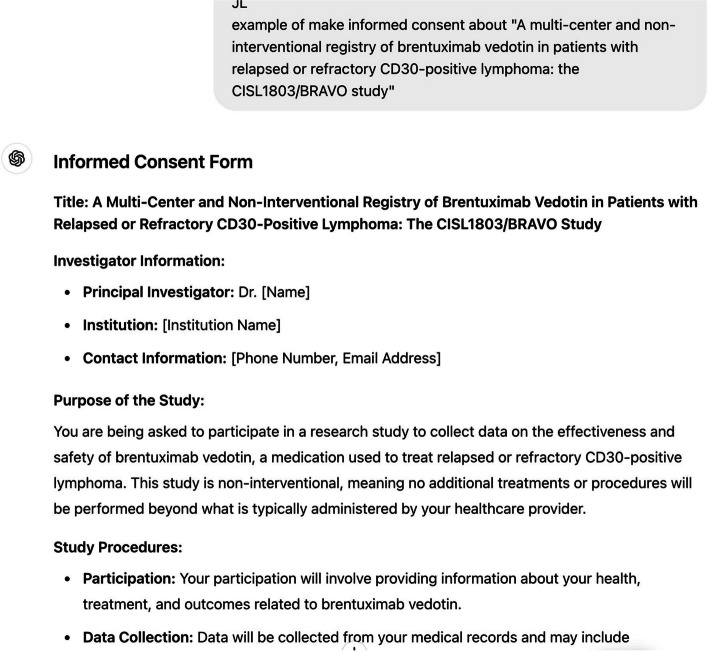
Informed consent form generated using GPT for a clinical study on brentuximab vedotin in patients with CD30-positive lymphoma

**Fig. 2:**
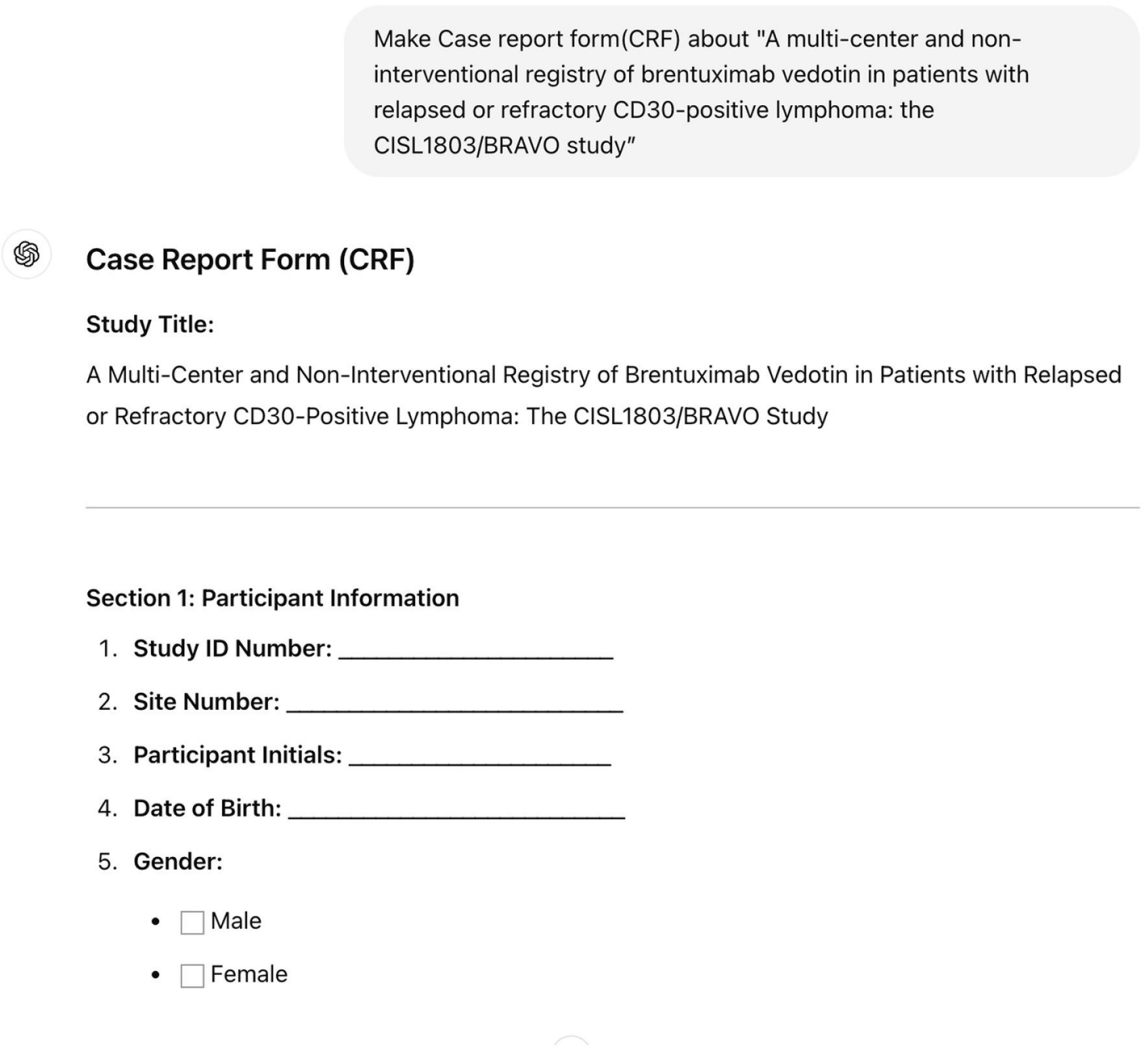
Case report form (CRF) generated using GPT for a clinical study on brentuximab vedotin in patients with CD30-positive lymphoma

**Fig. 3:**
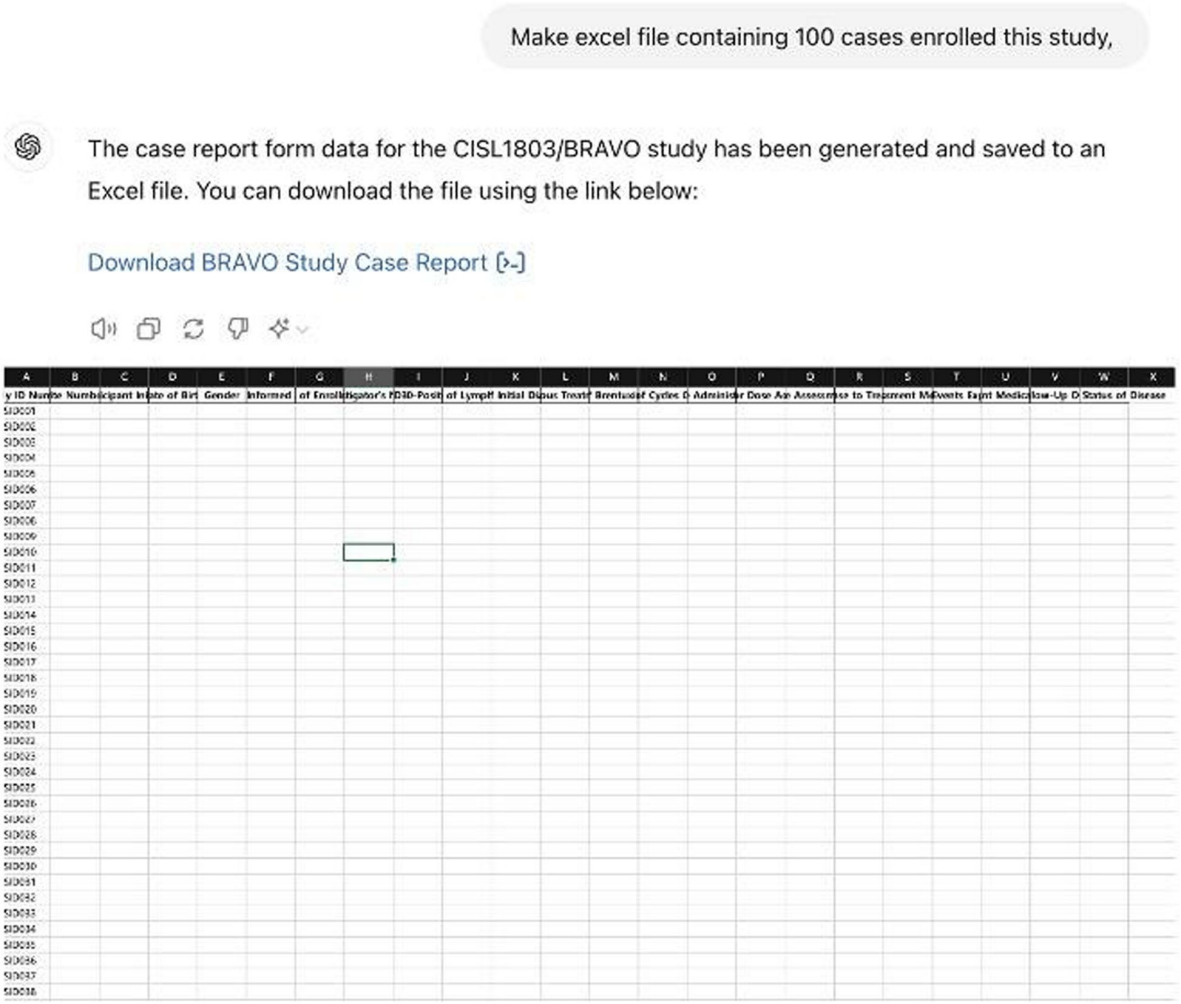
Database form Excel file generated using GPT for a clinical study on brentuximab vedotin in CD30-positive lymphoma

**Fig. 4:**
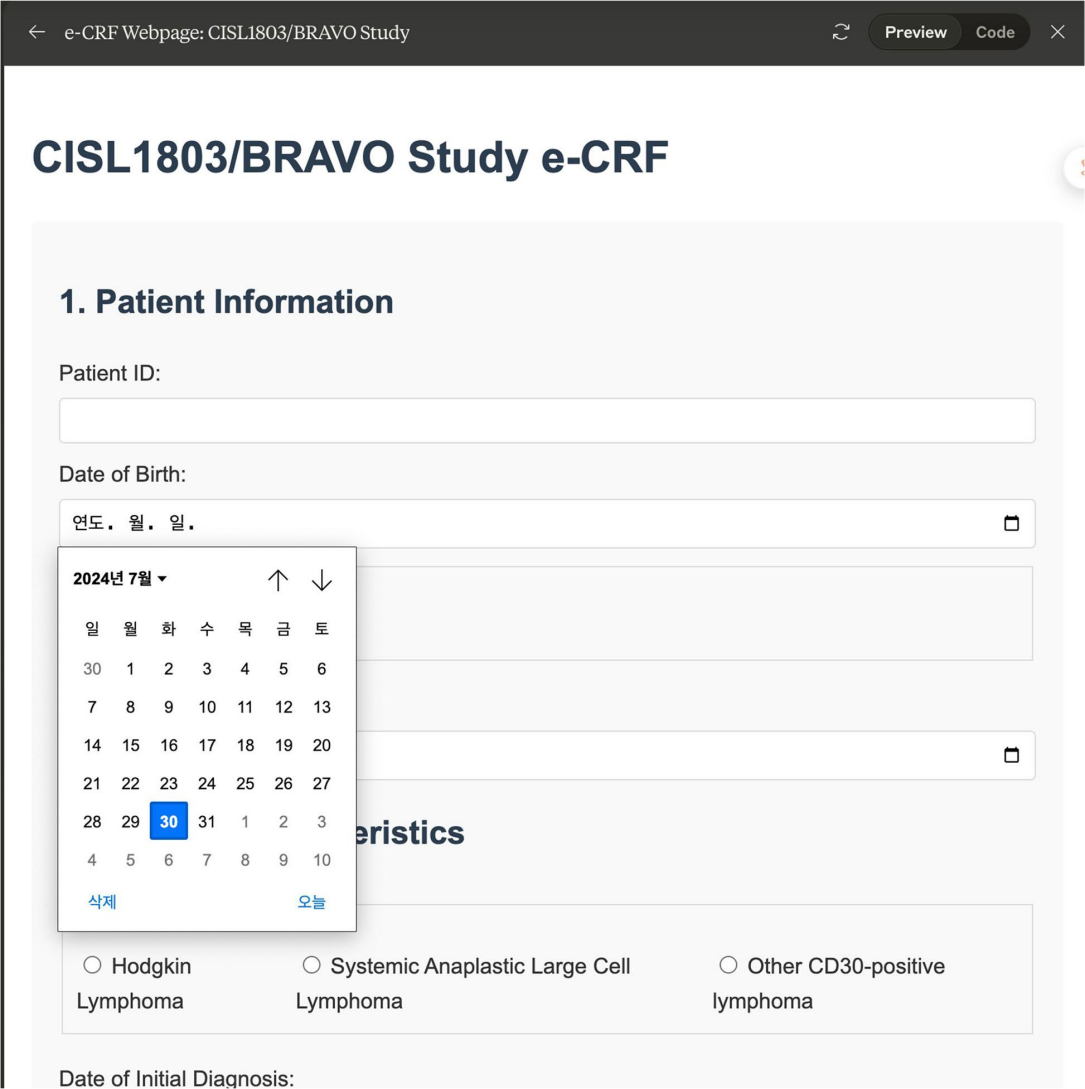
Webpage of E-CRF Excel file generated using Claude 3.5 sonnet for a clinical study on brentuximab vedotin in patients with CD30-positive lymphoma

**Fig. 5:**
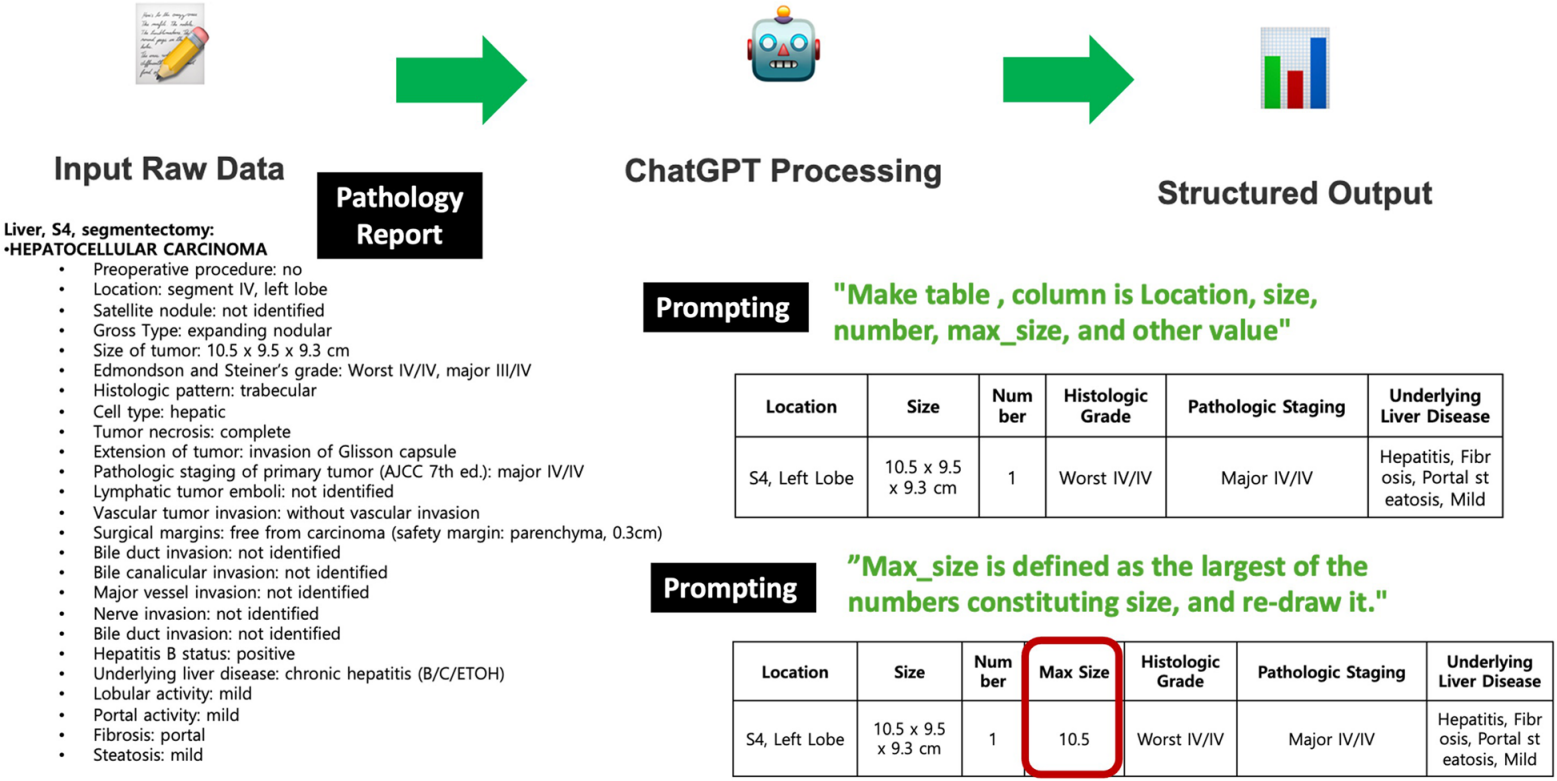
Data preprocessing (pathologic report parsing) process generated using GPT4o. The pathology data, including tumor location, size, histologic grade, and underlying liver disease, are organized into a clear, easily interpretable format. This facilitates time-saving for researchers and clinicians by efficiently converting complex medical data into a standardized table format

**Fig. 6:**
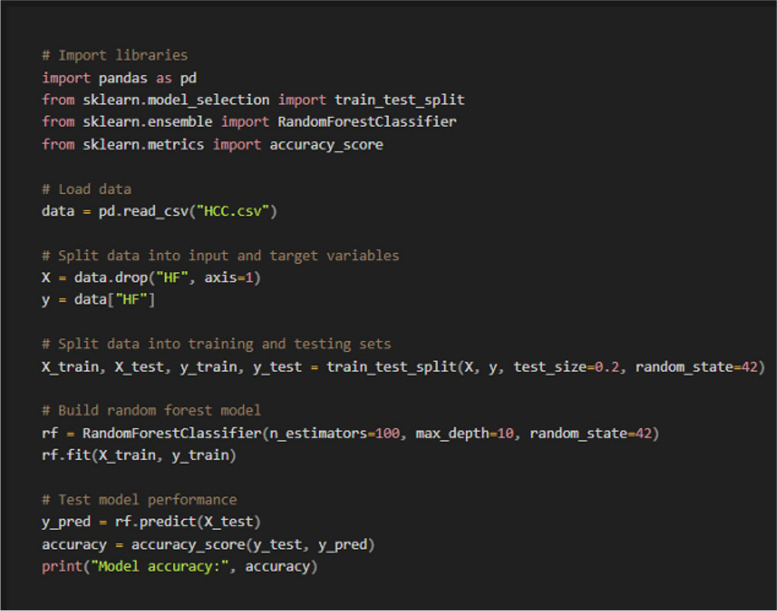
Python code generated using GPT4o for data analysis (Machine learning model)

- Informed consent (Fig. 1)
  - ◦Prompt: Make informed consent about "A multi-center and non-interventional registry of brentuximab vedotin in patients with relapsed or refractory CD30-positive lymphoma: the CISL1803/BRAVO study”[19]
- Case report form (Fig. 2)
  - ◦ Prompt: Make a case report form (CRF) about "A multi-center and non-interventional registry of brentuximab vedotin in patients with relapsed or refractory CD30-positive lymphoma: the CISL1803/BRAVO study”
- Database form for the research (Fig. 3)
  - ◦ Prompt: Make an excel file containing 100 cases enrolled this study
- E-CRF (Fig. 4)
  - ◦ Prompt: make it to E-CRF form, webpage

3) Data preprocessing with AI
  ◦ Pathology data parsing (Fig. 5)

- Prompt: Make table, column is Location, size, number, max_size, and other value

4) Data analysis

- Python code request (Machine learning model) (Fig. 6)
  - ◦ Prompt: Write Python code to make hepatic failure prediction model after liver resection, with a Random Forest model, from HCC.csv file the target column is “HF” all the other values are features and show the performance results
- Survival analysis

With a comprehensive understanding of the dataset, researchers can generate Kaplan–Meier or Cox proportional hazard survival plots by specifying the target variable and selecting relevant covariates. Using additional graphical packages, researchers can produce more sophisticated survival curves that are tailored to their specific analytical needs.

**Fig. 7:**
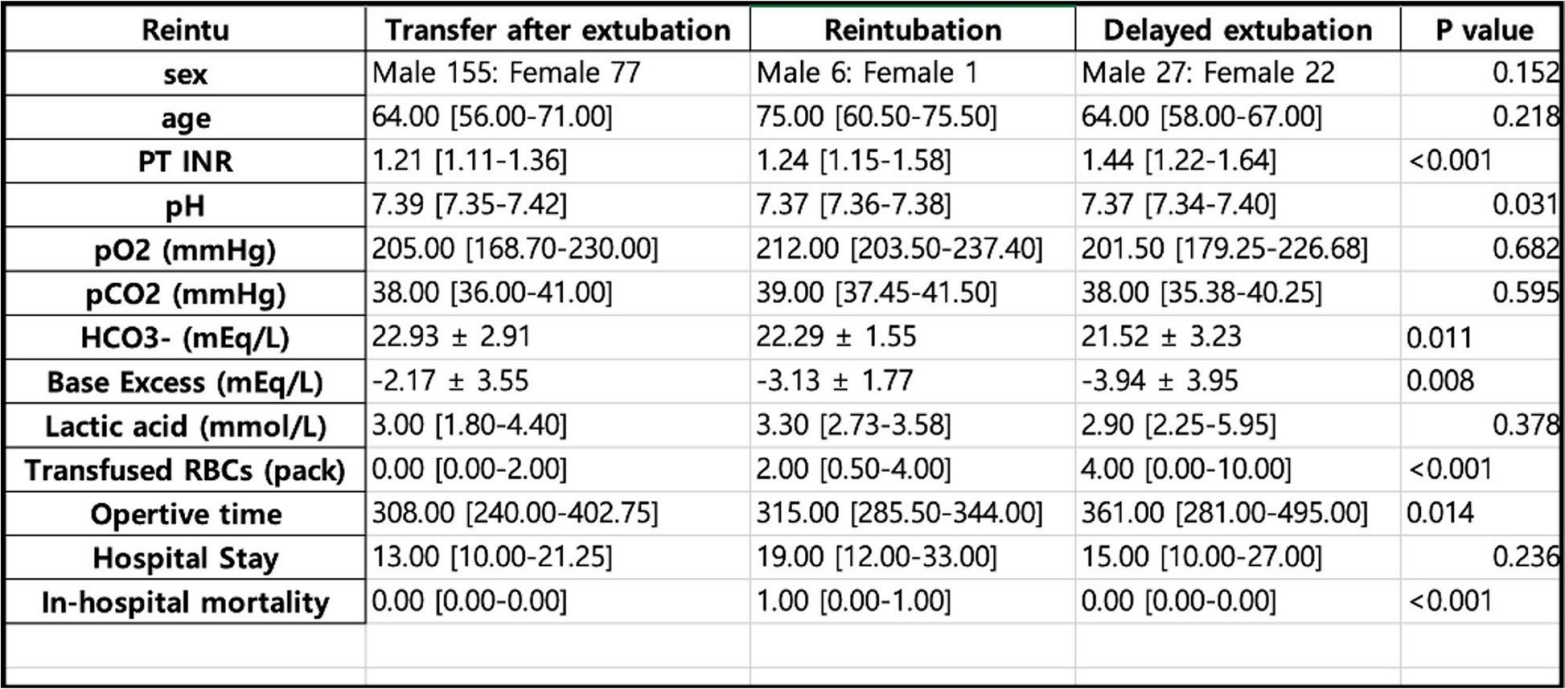
Example of three-group comparison table generation using GPT4o in the risk factor of reintubation after liver transplantation study

- ◦Prompt: Make a Survival curve based on uploaded data. RF survival is the survival time, and AllYes1No0 among 1|Recurrence is Recurrence. Excluding No, DeathYes1No0, RecurrencerelatedYes1No0, excluding RF_survival and columns 54–56, perform a Cox proportional hazards regression analysis on the remaining variables to obtain the factors and odds ratios that affect recurrence. If possible, also draw a recurrence-free survival graph.

5) Data visualization
  Table generation (Fig. 7)

ChatGPT can create tables after uploading the data. Common statistical analyses are conducted using simple prompts. The statistical methods used to compare groups are obtained by dividing continuous and categorical variables and then combining them into tables. However, it is possible to run statistics and output them in a table simultaneously using the advanced data analysis function of the GPT. Furthermore, if a normality test is added, as shown in the prompt below, a table for the paper can be output immediately without secondary processing.

**Fig. 8:**
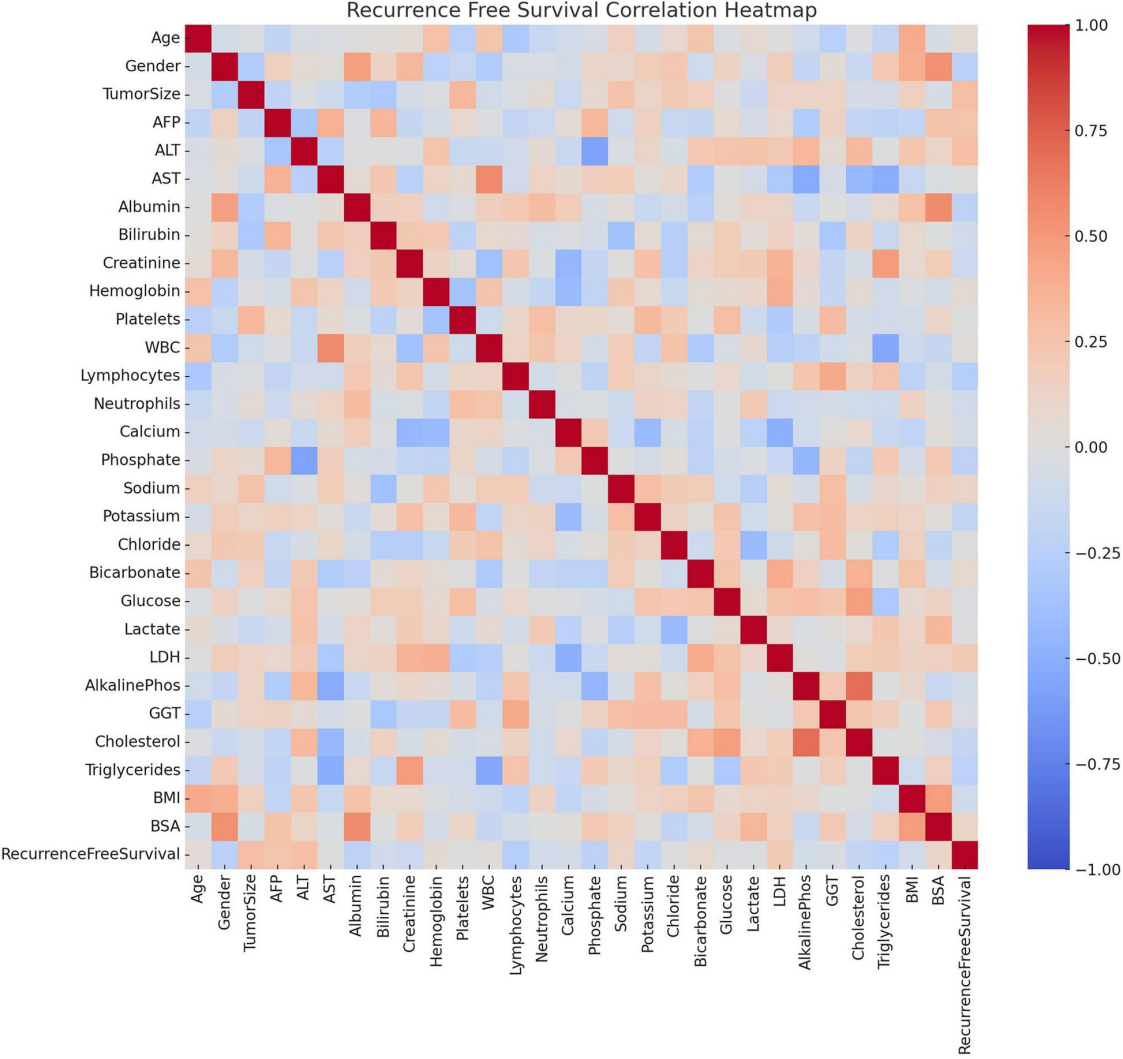
Example of figure generation using GPT4o in the study to identify the factors affecting recurrence after liver transplantation in patients with hepatocellular carcinoma, analyzing each variable in the form of a heatmap

- ◦Prompt: Using this data, create a table comparing the items for the three groups. The column "Reintu" contains the group information: 0 is "ICU Transfer after extubation group," 1 is "Reintubation group," and 2 is "Delayed extubation group." Create a comparison table for the three groups and add the P-value in the last column. Place the P-values after each group comparison in the last column. Write the P-value up to two decimal places. For continuous variables, if they appear normally distributed, write the mean and standard deviation; if not, write the median [IQR]. For categorical variables, write the results of the chi-square test.
• Figure generation (Fig. 8)

GPT offers the possibility of creating figures to illustrate the results of your paper using simple prompts. ChatGPT has over 300 built-in Python packages, which can be used to take advantage of the Matplotlib library to upload data and plot figures, such as the one below.

- ◦ Prompt: It is data of HCC patients. To identify the factors affecting recurrence-free survival, analyze each variable in the form of a heatmap and plot it.

### Manuscript writing step

1. Introduction

An individual who has written several academic papers knows the introduction section should include several elements:

- ◦ A summary of existing research
- ◦ Unresolved gaps in existing research
- ◦ The clinical implications and significance of the study
- ◦ Why I want to do this study

When writing a manuscript on the “Hepatic failure prediction model after liver resection,” it is advisable to request that collaborators draft an initial outline for the introduction section of the study. Subsequently, the outline can be refined by incorporating additional content:

- ◦ Risk of postoperative liver failure, clinical significance, incidence rate
- ◦ Summary of existing predictive model predictor studies
- ◦ Limitations of those studies (low predictive value, difficulty in clinical application)
- ◦ This study uses machine learning models to create a model to predict liver failure after liver resection.

- Prompt: Make Introduction session “hepatic failure prediction model after liver resection” including [ ], 2000words, professional English

The introduction should be drafted according to the specified prompt style using the outline provided as a guide. The style does not need to conform strictly to perfect English, word count, or any particular stylistic considerations. Moreover, the outline mentioned should be included in the text box. The initial draft should be composed using the specified writing style. If necessary, it may be reviewed and supplemented with additional content, including prompts. Additionally, in instances where it is beneficial to discuss the pros and cons of traditional predictive models or variables, requesting this information in a tabular or modified format is advisable. This approach aids in composing a more structured paper.

- Prompt: Pros and cons of previous prediction model, make tabular form.
- Prompt: Describe several Machine learning models to predict HF after liver resection. With pros and cons. Which model is the best model for predicting hepatic failure? Describe the reason

After formatting the table to meet one’s requirements, it should be requested to reform its contents in a manner suitable for academic publication. The following prompt can be provided: "Please describe this table in the format of a scientific article introduction, using professional academic English." The AI converts the contents of the table into structured text, presenting each element as a distinct paragraph. The following command should be issued: "Summarize the content into a single paragraph and explain the reason for selecting the random forest model." This method organizes and adds the desired content, allowing for modifications paragraph by paragraph. One can craft structured paragraphs and progressively complete the entire document by incorporating content block by block.

### Magic words

- ▪ Complete

This term is used to provide a topic for a paragraph and request its completion. This implies setting a table of contents and completing paragraphs accordingly.

- ▪ Develop

This prompt is useful for concluding and expanding a paragraph to include more content. This is particularly effective when requesting the initial output and developing writing further.

- ▪ Including

As in the previously mentioned example, the prompt requests the author to list the desired content in bullet points and then incorporate it using "including.” This approach provides generative AI based on the authors’ clear guidelines.

- ▪ Act as

Assigning a specific role to GPT can result in more professional responses. For example, starting a prompt with "Act as" followed by roles such as Editor or Scientific Medical Researcher can be highly beneficial for generating expert-level answers [20]. This approach is particularly effective when reviewing academic papers; assigning the reviewer role to a targeted journal can significantly aid in drafting insightful review comments.

2) Methods

The Methods section provides an outline of the study. The study design, variables, and topics should be described because the topic is chosen from a prompt. The outline is not the author's but ChatGPT’s own list. Therefore, it should be checked against one's own ideas and select those that are agreed upon; if not, further modifications can be made using the complete and developed commands to create the paragraphs. When inquiring about a study methodology, GPT may recommend a methodology or a statistical method. Requesting a method or table to help convey the intended message of the author would be appropriate. One may upload a PDF of related research and request that the methodology section define the proper approach to the topic.

Prompt: I want to make a research article comparison of A treatment vs B treatment. How can I perform this? Describe the research methods in the form of a scientific article, methods format.

- Prompt: Please choose the appropriate method for this study between VAE and GAN
- Prompt: I want to perform survival analysis, can you analyze this data and recommend an appropriate statistical method?

In addition, the GPT has expanded from an LLM to a multimodal model. While conducting research, it is often difficult to describe the method section. However, sometimes, a detailed figure of the research flow can explain the entire study well. In such cases, the figure can be uploaded and converted into text to complete the Methods section. However, it is necessary to ensure that accurate information is contained within the figure and consult Google search or the academic advisor to verify that there are no methodological issues with the recommended approach before using it.

3) Results

The results section is most appropriate for applying generative AI since it primarily involves an objective description of outcomes rather than subjective interpretations. Researchers, particularly in non-English-speaking countries, find it beneficial to upload statistical results or tables that convey objective facts in professional and academic English. Furthermore, when challenges arise in articulating the results, one may prompt GPT to draft an outline by stating, "We are trying to write a paper on [Your own research topic]. Please recommend a list of figures or tables to include in the Results section to support this.” Subsequently, one can create tables or figures or utilize other programs to develop these visual aids and upload them to GPT for detailed results.

“Describe this table in scientific article results format, Professional English.” Using this prompt, one can efficiently obtain a paragraph typical of a results section. However, if the study highlights a significant variable, including and specifying this in the prompt is crucial. For example, if the results indicate significant differences in complications between two treatments, one should instruct, "As the results of complications between the two treatments showed significant differences, emphasize this in the description" during the revision process to ensure the output meets the desired emphasis.

4) Discussion

The discussion section is pivotal in ethical and healthy research, granting roles such as academic researcher, medical researcher, and editor. AI can be instructed to write with reference to the previous content and may compose a discussion following the general format of the paper. However, such an approach yields a product that fails to reflect personal insights; thus, it cannot be deemed original work. A scientific article represents the written expression of thought process and must encapsulate the personal reflections of the author on the research findings. Lastly, while the purpose of generative AI is to streamline time-consuming and labor-intensive tasks like writing, data collection, and analysis, reliance on GPT for overall flow and logical development is not advisable.

For the draft, by referring to the previous content, dividing the clinical significance of the study, its strengths and limitations, and the valuable points despite these limitations, occasionally poses questions that provide unexpected insights. Overall, authors are advised to write to reflect their thoughts. The organization of insights into the discussion adopts an adaptive format. Including this list and encouraging the adoption of the discussion format allows for a draft that captures these thoughts effectively. This type of LLM proves invaluable for researchers who are not native English speakers.

### Review and formatting step

As mentioned earlier, when writing a paper review, one can assign a reviewer role to AI and request a review report. However, a simple review report is provided if a simple prompt is requested. Therefore, if it is requested in a critical tone and review points are asked for in a numbered or bullet list, one can draft a review report that includes criticisms. However, uploading articles requested for review to a GPT can raise copyright issues; thus, ensuring that the articles do not violate the guidelines of the journal is crucial. Additionally, GPTs may be unable to use uploaded data or chat transcripts for training; therefore, they should be adjusted accordingly. GPTs are useful for writing responses to reviews. The issues flagged in the manuscript by the reviewer can be documented, and a response can be provided. If the GPT takes many tokens to run, the quality of the subsequent output will be poor, and it is beneficial to ask questions by selecting only the relevant parts; maintaining a polite tone helps in obtaining good responses.

Revising rejected papers to comply with varying journal author guidelines remains a significant challenge for researchers. Previously, incorporating the full text of a paper was problematic, but current technology allows for the complete reading of papers. However, incorporating author guidelines and subsequent editing does not guarantee flawless outcomes. As highlighted, consuming numerous tokens by reading the entire paper depletes the token reserve necessary for creation and output, compromising the output quality.

In response to this issue, the author guidelines can be consulted, and a request for a succinct report to pinpoint the discrepancies can be made. This approach ensures efficient token usage in GPT, as it merely assesses adherence to the guidelines and generates a brief report, leading to a productive output.

### Ethical use of generative AI: plagiarism and AI detector

Using generative AI in academic writing raises several ethical concerns, particularly regarding plagiarism. Generative AI can produce content that closely mimics human writing, which can lead to unintentional plagiarism when used without proper attribution. The technology understands the context and generates content rather than directly copying existing material; thus, concerns about plagiarism are not as significant as expected. Nevertheless, researchers using AI-generated text must acknowledge the role of AI in their work and ensure adherence to proper citation practices. Careless use without adequate fact-checking can undermine the reliability of the research and must be approached with caution. Recently, there have been paper retractions where phrases like "I’m a language model" commonly generated by AI at the beginning of texts, were included without adequate review, leading to detection. Such oversights may be attributed to careless authors, inattentive reviewers, and lackadaisical editors, resulting in severe consequences. Therefore, statements generated by the LLMs must be fact-checked and reviewed by the authors. Several tools are available to assist with this process. These tools can help prevent plagiarism and the errors that led to the aforementioned retractions. SciSpace offers a paraphrasing feature, and assistance can be obtained from Grammarly or Quilbot, which are paid English-language proofreading tools. These tools have been around for a while but have recently enhanced their performance by incorporating AI technology. Moreover, it is important to recognize that papers in the closed DB that are not publicly available in full text are based on abstracts, which may limit the level of detail and depth of analysis. Additionally, hallucinations are challenging to prevent entirely. Therefore, fact-checking is indispensable.

Several journals and schools have recently used AI detectors to prevent AI from writing assignments. The notable tools include GPTzero and ZeroGPT. These tools have continued to improve and are highly effective at identifying AI-generated texts. In recent articles, the frequency of words such as "delve," which are favored by GPT, has increased. This sudden increase suggests that many researchers use GPT to write academic papers [21].

AI detectors can be circumvented to a certain extent through the use of simple prompts. A command can prevent the AI detector from triggering, indicating a decrease in the AI writing proportion. Nevertheless, AI detectors often exhibit a high false-positive rate, complicating their use as a criterion for retracting papers [22]. In certain instances, even papers from 2019, before the availability of GPTs, were misidentified as authored by GPTs. In other instances, papers were erroneously labeled as solely produced by GPTs despite only being used for proofreading. Consequently, many chief editors of scientific journals have acknowledged the impracticality of perfect detection and advice authors to transparently attribute AI usage rather than prohibit it. Current concerns about the use of AI tools, stemming either from the limitations of AI detectors or ethical apprehensions, are understandable but are unlikely to pose long-term issues. Instead of dwelling on these concerns, it would be more advantageous to leverage capabilities of AI, especially for expediting labor-intensive tasks such as data cleaning, analysis, and paper writing.

### Strategies for integration to clinical study

Generative AI is an efficient tool for organizing and expressing ideas, comparable to word processors for drafting or specialized software such as SPSS and R for statistical analysis. However, a scientific article fundamentally represents the thought process of an author. Thus, while AI-generated content can be helpful, it must accurately reflect the insights and conclusions of the researcher. Additionally, it is crucial not to finalize the content without thoroughly reviewing and verifying AI-generated sentences, as blindly pasting them can lead to problems. Therefore, fact-checking using various tools and tips is essential to ensure the accuracy of the sources in an article and to maintain the credibility of the author. Moreover, it is important to remember that AI tools cannot do more than is asked; thus, frustration is unnecessary.

In conclusion, LLM serves as a powerful tool for efficiently organizing and expressing ideas; however, it is essential to recognize its limitations. Academic paper writing remains a serious process that demands the author's critical analysis and intellectual contributions. AI-generated text should be meticulously reviewed to reflect the authors’ insights and understanding. Therefore, although using AI can significantly enhance productivity, it remains a responsibility to critically engage with and refine its output to ensure the quality and credibility of academic work.

## Data Availability

No datasets were generated or analysed during the current study.
