# Correction: Strategies for integrating ChatGPT and generative AI into clinical studies

**DOI:** 10.1007/s44313-025-00058-6

**Published:** 2025-01-21

**Authors:** Jeong‑Moo Lee

**Affiliations:** Department of Surgery, Division of HBP Surgery, Seoul National University Hospital, Seoul National University College of Medicine, 101 Daehak‑ro, Jongno‑Gu, Seoul, 03080 Republic of Korea

**Correction: Blood Res 59, 45 (2024)**

**https://doi.org/10.1007/s44313-024-00045-3**

Following publication of this article [1], we became aware that the in-text citations were taken from an earlier draft rather than the final version due to a typesetting error.

The reference list remains accurate and unchanged.

The incorrect version has been included as supplementary file in this erratum for the readers’ reference and the original article has been corrected.

The publisher would like to apologize for any confusion this may have caused.

## Supplementary Information

Supplementary Material 1.
